# Prevalence and Predictors of Nonadherence to Direct Oral Anticoagulant Treatment in Patients with Atrial Fibrillation

**DOI:** 10.1055/a-2161-0928

**Published:** 2023-09-27

**Authors:** Sabine F. B. van der Horst, Tim A.C. de Vries, Gordon Chu, Roisin Bavalia, Helen Xiong, Kayleigh M. van de Wiel, Kelly Mulder, Hanne van Ballegooijen, Joris R. de Groot, Saskia Middeldorp, Frederikus A. Klok, Martin E.W. Hemels, Menno V. Huisman

**Affiliations:** 1 Department of Thrombosis and Hemostasis, Leiden UMC, Leiden, The Netherlands; 2 Department of Clinical and Experimental Cardiology and Cardiothoracic Surgery, Amsterdam UMC location University of Amsterdam, Heart Center, Amsterdam, The Netherlands; 3 Amsterdam Cardiovascular Sciences, Heart Failure and Arrhythmias, Amsterdam, The Netherlands; 4 Department of Cardiology, Hospital Rijnstate, Arnhem, The Netherlands; 5 Department of Internal Medicine, Alrijne Hospital, Leiden, The Netherlands; 6 Department of Vascular Medicine, Amsterdam UMC location University of Amsterdam, The Netherlands; 7 GGD Amsterdam, Amsterdam, The Netherlands; 8 IQVIA Netherlands, Amsterdam, The Netherlands; 9 Department of Internal Medicine, Radboudumc, Nijmegen, The Netherlands; 10 Department of Cardiology, Radboudumc, Nijmegen, The Netherlands

**Keywords:** direct oral anticoagulant, anticoagulants, medication adherence, atrial fibrillation

## Abstract

**Background** For most patients with newly diagnosed atrial fibrillation (AF), direct oral anticoagulants (DOACs) are preferred over vitamin K antagonists. However, there is concern that the lack of monitoring may impair therapy adherence and therefore the anticoagulant effect.

**Objective** To assess 1-year DOAC nonadherence in patients with AF and a treatment indication of at least 1 year in the Dutch health care setting, and to identify predictors of nonadherence.

**Methods** We performed a near-nationwide historical cohort study in patients with a novel DOAC indication for AF. Data were obtained from a pharmacy database, covering 65% of all outpatient prescriptions dispensed in the Netherlands. The 1-year nonadherence was assessed by the proportion of days covered; the threshold was set at <80%. Robust Poisson regression analyses were performed to identify predictors of nonadherence.

**Results** A total of 46,211 patients were included and the 1-year nonadherence was 6.5%. We identified male sex (risk ratio [RR] 1.23, 95% confidence interval [CI]: 1.15–1.33), younger age (age ≥60 to <70 years: RR: 1.15, 95% CI: 1.00–1.33, age <60 years: RR: 2.22, 95% CI: 1.92–2.57; reference age ≥85 years), a reduced DOAC dose (RR: 1.10, 95% CI: 1.00–1.22), a twice-daily dosing regimen (RR: 1.21, 95% CI: 1.12–1.30), and treatment with apixaban (RR: 1.16, 95% CI: 1.06–1.26, reference rivaroxaban) or dabigatran (RR: 1.25, 95% CI: 1.14–1.37) as independent predictors of 1-year nonadherence.

**Conclusion** One-year nonadherence to DOACs was low yet relevant in patients with AF newly prescribed a DOAC. Understanding the predictors for nonadherence may help identify patients at risk.

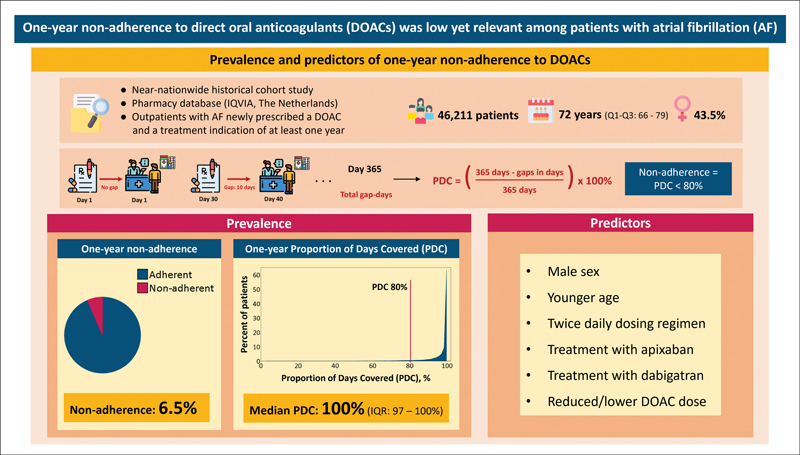

## Introduction

Direct oral anticoagulants (DOACs) are indicated for the prevention of thrombotic complications in patients with atrial fibrillation (AF) and are preferred over vitamin K antagonists (VKAs) as anticoagulant treatment in most anticoagulation-naïve patients with this arrhythmia. 1 This is because DOACs, compared to VKA targeted at an international normalized ratio of 2.0 to 3.0, were are at least as effective and had a better safety profile. 2 3 4 5 In a meta-analysis, DOACs reduced the endpoint of stroke or systemic embolism by 19%, showed a strong trend toward fewer major bleedings, and mortality was significantly lower as compared to VKAs. 6 Moreover, DOACs have an improved ease of use due to the fixed dosing regimen, obviating the need for routine laboratory monitoring.

Yet, there is concern that the lack of monitoring may impair therapy adherence. Two systematic reviews and meta-analyses suggest that roughly 30% (range: 5–59%) of patients treated with a DOAC are nonadherent within the first year of treatment, defined as medication possession ratio (MPR) or proportion of days covered (PDC) of <80%. 7 8 As a consequence of therapy nonadherence, the risk of thromboembolic complications could potentially increase. 9 The increased thrombotic risk might be enhanced by the shorter plasma elimination half-life and concomitant limited duration of anticoagulant effect of DOACs compared to VKAs. Identifying the patients at risk of becoming nonadherent and implementing strategies to reinforce adherence may therefore further optimize anticoagulant care. Since several studies show that education, reminders, and active monitoring improve adherence in patients using a DOAC, it is important to identify those patients who could benefit from such interventions. 10 11 Previous research has focused on elucidating predictors for nonadherence, and adherence was found to vary significantly between patients using various DOACs. There is evidence that patients treated with twice-daily dosed DOACs, especially dabigatran, are less likely to adhere to treatment, with studies showing that nonadherence (PDC/MPR < 80%) is present in up to half of these patients. 12 13 14 15 16 17

Nonetheless, most prior studies on DOAC nonadherence in patients with AF did not consider the indicated treatment duration when assessing the prevalence of 1-year nonadherence. Among patients with AF, temporary treatment indications with a DOAC, such as cardioversion or ablation, are common. When these patients are included in 1-year nonadherence assessments, PDC decreases leading to an inadvertent increase in the nonadherence prevalence. To get a better understanding of nonadherence among AF patients who receive long-term treatment with a DOAC, it is important to evaluate 1-year nonadherence in patients who actually have a treatment indication for at least 1 year.

To this end, we performed a historical cohort study using dispensing data from a Dutch representative nationwide pharmacy database. We aimed to determine the prevalence of therapy nonadherence to DOACs in outpatients with AF newly initiated on a DOAC for at least 1 year, and to identify potential predictors of such nonadherence at the time DOAC treatment was initiated in these patients.

## Methods

### Study Design and Data Source

We performed a historical cohort study using data from IQVIA's Xtrend Real-World Data Longitudinal Prescription database (Xtrend-LRx, Amsterdam, The Netherlands). This dataset comprises prescription records, including patient characteristics (age, sex), dispensing details (pharmacy, prescription, and dispensing data) and medication specifics (name, dose, strength, therapy duration). All data in the database were provided by pharmacies and were first pseudonymized by a third party before being incorporated into the dataset. The database covers approximately 65% of all prescriptions filled by outpatients in the Netherlands, represented by retail pharmacies, outpatient hospital pharmacies, and dispensing general practitioners ( Supplementary Fig. S1 ). Per October 1, 2014, patients were given a unique identifier ensuring longitudinal follow-up for each patient, even if those who collected prescriptions at different affiliated pharmacies during the study period. Data of pharmacies that failed to provide uninterrupted data for the entire study duration were excluded from the dataset.

### Study Population

All patients who filled their first DOAC prescription between November 1, 2014 and October 31, 2019 were identified from the Xtrend-LRx database. The European Pharmaceutical Market Research Association Anatomical Classification System (EPHMRA ATC, 2018) was used to identify the DOACs of interest: apixaban (ATC B01AF02), dabigatran (ATC B01AE07), edoxaban (ATC B0AF03), and rivaroxaban (ATC B01AF01). 18 Patients were eligible for inclusion if they were newly starting a DOAC (i.e., no DOAC prescription fill within the 12 months prior to the initial fill) and had a treatment indication of at least 1 year (i.e., a prescription fill of the same DOAC 12 months after the initial fill). To this end, a look-back and look-forward period of 12 months was implemented and only patients with an initial prescription fill between November 1, 2015 and October 31, 2019 were included. Patients who met any of the following criteria were excluded: (1) those aged <18 years; (2) patients who collected more than one type of oral anticoagulation at the time of the initial DOAC prescription fill; and (3) patients with an initial DOAC treatment indication other than AF or with a dosing regimen not approved for AF. A decision tree model was developed, based on dosing regimen, treatment duration, and pretreatment with low-molecular-weight heparin, to estimate the most probable indication for treatment with a DOAC ( Supplementary Table S1 , Supplementary Fig. S2 ). 19 20 21

### Baseline Characteristics and Outcome

We collected baseline data on demographics (i.e., age and sex) and on the initially filled DOAC prescription (i.e., type, dose, dosing frequency, clinical field of the prescriber, prescription date, number of pills). DOAC dosing regimens were classified as either standard dose, reduced dose based on clinical characteristics (for apixaban, edoxaban, and rivaroxaban), or lower dose (dabigatran 110 mg twice daily), in accordance with The European Heart Rhythm Association Practical Guide ( Supplementary Table S1 ). 19 Baseline was defined as the day of the first filled DOAC prescription.

The primary outcome of the study was 1-year nonadherence, defined as <80% of days per year covered by filled prescriptions of the index DOAC. We calculated the PDC as follows: total number of days covered by index medication, divided by 365 days. The first DOAC prescription was defined as index medication and the date the prescription was filled as the index date. The estimated duration of DOAC prescriptions was calculated based on the prescription date, days of supply, and the dosing regimen. Subsequent dispensing data were assessed to identify gaps in DOAC treatment. If the prior prescription ended prior to the subsequent dispensing date, it was considered a gap. An overlap, defined as a prior prescription extending past the subsequent dispensing date resulting in a surplus, was considered to carry-over to the subsequent prescription.

### Statistical Analysis

The demographic and clinical characteristics of the study population at baseline were expressed as either frequencies and percentages, means with standard deviations (SD), or medians with interquartile intervals (Q1–Q3), for the overall group, adherent patients (PDC ≥ 80%), and nonadherent patients (PDC < 80%). The Kruskal–Wallis test and the chi-square test were used to compare the PDC and nonadherence rates across the different DOACs, respectively. A *p* -value less than 0.05 was considered statistically significant. The dataset did not contain any missing values.

Univariable and multivariable robust Poisson regression analyses were performed to identify predictors of nonadherence and to calculate risk ratios (RRs) and their corresponding 95% confidence intervals (95% CIs). The following potential predictors of nonadherence were selected based on existing evidence and expert opinion: age, sex, type of DOAC, dosing regimen, and dose reduction. To avoid multicollinearity, we performed two separated multivariable regression analyses, with either the specific DOACs used or dosing regimen (i.e., once or twice daily [QD/BID]) as potential predictors for nonadherence. 22 In the analysis by DOAC type, rivaroxaban was considered as a reference category due to the once-daily dosing regimen and group size. To allow for a nonlinear relationship with the outcome of interest (nonadherence), age was modelled using a restricted cubic spline function. The knot locations were kept standard and were based solely on the number of knots of the optimal fit, defined as the model with the lowest Bayesian Information Criterion value. Age was categorized based on these knot locations and clinical applicability. All statistical analyses were conducted using R (version 4.1.2.) within RStudio (version 2022.07.2) or SPSS (version 25.0).

## Results

### Patient Selection and Baseline Characteristics

**Fig. 1:**
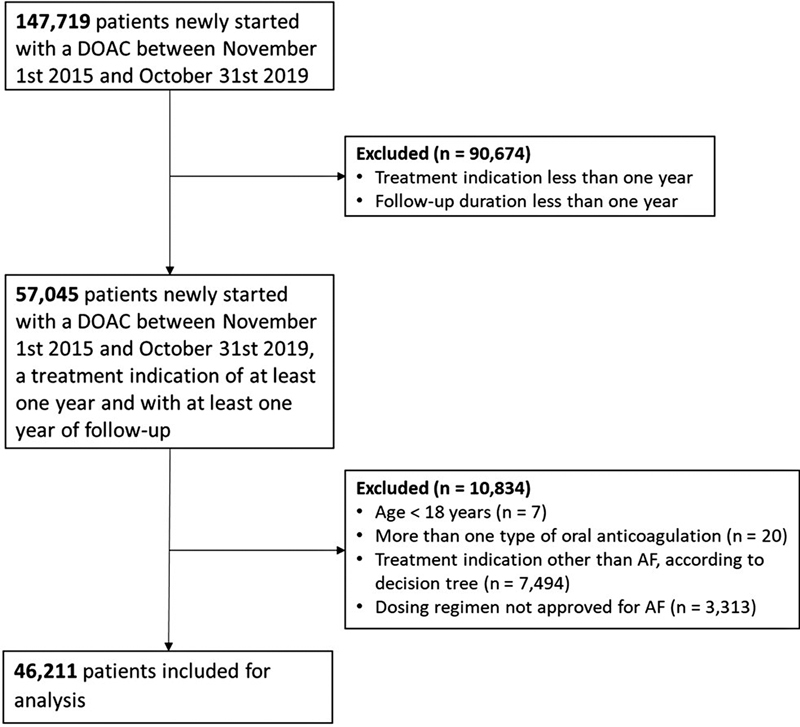
Flow diagram of patient inclusion and exclusion.

Overall, a total of 147,719 patients filled their first DOAC prescription between November 1, 2015 and October 31, 2019. After excluding patients with a treatment indication or follow-up duration of less than 1 year ( *n* = 90,674), those aged <18 years ( *n* = 7), those who were prescribed more than one type of anticoagulation at the time of the initial DOAC prescription fill ( *n* = 20), those with a treatment indication other than AF based on the decision tree ( *n* = 7,494), and those with a dosing regimen not approved for AF ( *n* = 3,313), a total of 46,211 patients were included in the analysis ( Fig. 1 ). The baseline characteristics are presented in Table 1 . The median age was 72 years (Q1–Q3: 66–79 years) and the majority of patients were male (56.5%). The most commonly initially collected DOAC was rivaroxaban (35.8%), followed by apixaban (33.3%), dabigatran (24.1%), and edoxaban (6.8%). A reduced or lower DOAC dose was prescribed to 20.6% of patients.

**Table 1:** Clinical characteristics of the enrolled patients

Characteristics	Overall ( *n* = 46,211)	Adherent ( *n* = 43,205)	Non-adherent ( *n* = 3,006)
Age at index in years, median (Q1 - Q3)	72 (66–79)	73 (66–80)	70 (61–78)
Age category at index in years, *n* (%)			
<60	5,468 (11.8)	4,805 (11.1)	663 (22.1)
≥60 to <70	12,057 (26.1)	11,300 (26.2)	757 (25.2)
≥70 to <85	23,429 (50.7)	22,133 (51.2)	1,296 (43.1)
≥85	5,257 (11.4)	4,967 (11.5)	290 (9.6)
Male, *n* (%)	26,126 (56.5)	24,235 (56.1)	1,891 (62.9)
Type of DOAC, *n* (%)			
Apixaban	15,757 (34.1)	14,710 (34.0)	1,047 (34.8)
Dabigatran	11,422 (24.7)	10,578 (24.5)	844 (28.1)
Edoxaban	3,437 (7.4)	3,243 (7.5)	194 (6.5)
Rivaroxaban	15,595 (33.7)	14,674 (34.0)	921 (30.6)
DOAC dosing frequency, *n* (%)			
Once daily	19,032 (41.2)	17,917 (41.5)	1,115 (37.1)
Twice daily	27,179 (58.8)	25,288 (58.5)	1,891 (62.9)
DOAC dosing, *n* (%)			
Standard dose	36,700 (79.4)	34,292 (79.4)	2,408 (80.1)
Reduced/lower dose	9,511 (20.6)	8,913 (20.6)	598 (19.9)
Adherence			
Number of gaps, median (Q1 - Q3)	0.0 (0.0–1.00)	0.0 (0.0–1.0)	2.0 (1.0–3.0)
Gaps in days, median (Q1 - Q3)	0.0 (0.0–10.0)	0.0 (0.0–7.0)	116 (90.0–164.0)
PDC in %, median (Q1 - Q3)	100 (97–100)	100 (98–100)	68 (55–75)
Adherent, *n* (%)	43,205 (93.5)	43,205 (100)	0 (0.0)
Nonadherent, *n* (%)	3,006 (6.5)	0 (0.0)	3,006 (100)

Abbreviations: DOAC, direct oral anticoagulant; n, number; PDC, proportion of days covered; SD, standard deviation.

Note: Data are presented as mean (SD) or number (%).

### Nonadherence

The overall median PDC was 100% (Q1–Q3: 97–100%). Using a PDC threshold of <80%, 6.5% of the patients were nonadherent after 1 year of treatment. Nonadherent patients were younger (median age: 70 years vs. 73 years) and more often male (62.9 vs. 56.1%). Moreover, a twice-daily dosing regimen of DOACs (62.9 vs. 58.5%) was more common among nonadherent patients ( Table 1 ). The 1-year prevalences of nonadherence were 6.6, 7.4, 5.6, and 5.9% ( *p* < 0.001) for apixaban, dabigatran, edoxaban, and rivaroxaban, respectively ( Table 2 ).

**Table 2:** Proportion of days covered (PDC) and nonadherence by type of direct oral anticoagulant (DOAC)

	Overall	Apixaban	Dabigatran	Edoxaban	Rivaroxaban	*p* -Value
PDC in %, median (Q1 - Q3)	100 (97–100)	100 (97–100)	100 (96–100)	100 (98–100)	100 (98–100)	<0.001
Nonadherence, %	6.5%	6.6%	7.4%	5.6%	5.9%	<0.001

Note: *p* -Values were calculated by Kruskal–Wallis test (PDC) and chi-square test (nonadherence).

### Predictors of Nonadherence

In multivariable robust Poisson regression analysis, male sex (RR: 1.23, 95% CI: 1.15–1.33), age ≥60 to <70 years (RR: 1.15, 95% CI: 1.00–1.33; reference age ≥85 years), age <60 years (RR: 2.22, 95% CI: 1.92–2.57), dabigatran (RR: 1.25, 95% CI: 1.14–1.37, reference rivaroxaban), apixaban (RR: 1.16, 95% CI: 1.06–1.26), and a reduced or lower dose (RR: 1.10, 95% CI: 1.00–1.22) were independent predictors of nonadherence. When including dosing regimen instead of specific DOAC into the multivariable model, a twice-daily dosing regimen was an independent predictor of nonadherence (RR: 1.21, 95% CI: 1.12–1.30) ( Table 3 ).

**Table 3:** Univariable and multivariable logistic regression analyses to identify predictors of nonadherence

	Univariable analysis	Multivariable analysis by type of DOAC	Multivariable analysis by dosing regimen
	RR (95% CI)	RR (95% CI)	RR (95% CI)
Age—in years			
≥85	Reference (1.00)	Reference (1.00)	Reference (1.00)
≥70 to <85	1.00 (0.89–1.13)	1.01 (0.89–1.16)	1.02 (0.90–1.17)
≥60 to <70	1.14 (1.00–1.30)	1.15 (1.00–1.33)	1.17 (1.01–1.35)
<60	2.20 (1.92–2.51)	2.22 (1.92–2.57)	2.25 (1.95–2.60)
Sex, male	1.30 (1.21–1.40)	1.23 (1.15–1.33)	1.24 (1.15–1.33)
Type of DOAC			
Rivaroxaban	Reference (1.00)	Reference (1.00)	N.a.
Edoxaban	0.96 (0.82–1.11)	0.95 (0.82–1.10)	N.a.
Dabigatran	1.25 (1.14–1.37)	1.25 (1.14–1.37)	N.a.
Apixaban	1.13 (1.03–1.23)	1.16 (1.06–1.26)	N.a.
DOAC dosing frequency			
Once daily	Reference (1.00)	N.a.	Reference (1.00)
Twice daily	1.19 (1.11–1.28)	N.a.	1.21 (1.12–1.30)
DOAC dosing			
Standard dose	Reference (1.00)	Reference (1.00)	Reference (1.00)
Reduced/lower dose	0.96 (0.88–1.05)	1.10 (1.00–1.22)	1.13 (1.02–1.24)

Abbreviations: CI, confidence interval; DOAC, direct oral anticoagulant; N.a., not applicable; RR, risk ratio.

Note: Univariable and multivariable robust Poisson regression analyses were performed to identify predictors of nonadherence and to calculate risk ratios (RRs) and their corresponding 95% confidence intervals (95% CIs).

## Discussion

We performed this near-nationwide historical cohort study to determine the prevalence of therapy nonadherence to DOACs in outpatients with AF, and to identify potential predictors of such nonadherence at the time DOAC treatment was initiated.

Nonadherence is supposed to be a major concern among patients using DOACs. The absence of immediate and observable benefits from thromboembolic prophylaxis may lead to a lack of motivation to continue taking oral anticoagulation as prescribed. Additionally, the long-term treatment indication may make it difficult for patients to maintain adherence over time. The lack of need for routine monitoring may further contribute to nonadherence in patients using DOACs, since monitoring can serve as an active reminder for patients to take their anticoagulant. Moreover, missing one or a few doses may reduce the anticoagulant effect of DOACs more than it would of VKAs because of two reasons. First, the administered dose of VKAs is adjusted based on the measured anticoagulant activity, whereas for DOACs the anticoagulant is not routinely assessed. Second, DOACs have a more rapid onset and offset of effect than VKAs due to differences in their mechanism of action and elimination half-lives. Being able to identify patients at risk of becoming nonadherent to implement targeted adherence, reinforcing strategies may contribute to the optimalization of anticoagulant care and the prevention of thromboembolic events.

In the 46,211 AF patients newly prescribed a DOAC and receiving at least 1 year of treatment in the Netherlands, the prevalence of 1-year nonadherence was 6.5%. We identified male sex, younger age, a twice daily dosing regimen, treatment with apixaban or dabigatran, and a reduced or lower DOAC dose as independent predictors of 1-year nonadherence.

### Prevalence of 1-Year Nonadherence

The treatment nonadherence found in our study is in line with that of a previous pharmacy-based study in Swedish (nonadherence percentage: 5.5%, mean MPR: 96.0% ± 7.8) and Dutch AF patients (nonadherence percentage: 7.4%, mean MPR: 95.1% ± 10.1) using DOACs. 23 Interestingly, another study showed significantly lower nonadherence rates in cohorts of AF patients in the Netherlands (1-year nonadherence: QD/BID users 6%/9%; mean PDC: QD 96% ± 10, BID 95% ± 13) compared to cohorts in Italy (1-year nonadherence: QD/BID: 11%/12%) and Germany (1-year nonadherence: QD/BID: 18%/38%). 24 However, higher nonadherence estimates have been described in the literature as well. Even though median MPR (95.2%, interquartile range: 87.8–99.7%) was still high in a pharmacy-based Belgian study ( *n* = 766 patients), the percentage of nonadherent patients (13%) was higher compared to our study. 25 Other studies found 1-year mean PDCs/MPRs of around 85% and nonadherence percentages of approximately 25%, with (similar to our results) even worse adherence in patients prescribed dabigatran. 9 12 26 27 28 29

Given that follow-up duration and the criteria used to define nonadherence (PDC or MPR <80%) in both our study and the aforementioned studies were comparable, it is plausible that there are additional factors contributing to the variations in nonadherence estimations. First, the availability of a unique patient identifier allowed us to follow up patients even when they switched to another pharmacy. Second, the 12-month look-forward period enabled us to only include those patients with a treatment indication for the specific DOAC for at least 1 year. Most prior studies on nonadherence to DOAC therapy did not take into account the indicated treatment duration. As a result, patients without a DOAC indication for at least 1 year, but a need for anticoagulation prior to electrical cardioversion or ablation, may have been included in these studies and could have inadvertently increased the number of nonadherent patients. For instance, a large population-based study in the Netherlands ( *n* = 43,910) reported a nonadherence percentage of 24%. However, when excluding patients who completely discontinued treatment within the first year ( *n* = 23,098), the nonadherence rate was 3% with a mean PDC of 0.97. 29 Additionally, upon excluding patients who discontinued treatment (no refill 12 months after the initial prescription), our study population consisted of patients who were more likely to adhere to treatment, potentially leading to overestimation of adherence. Lastly, the observed discrepancies could potentially be attributed to differences in the methodologies utilized for gathering data on adherence. For instance, in the study by Banerjee et al, dispensing data (data on actual prescription fills) were lacking and adherence was estimated from the available prescription data. 12 Consequently, the results relating to adherence may be relatively less accurate when compared to our study.

Although the prevalence of 1-year nonadherence to DOACs of 6.5% may appear low, our findings indicate that a relevant proportion of patients with AF remain at risk of thromboembolic events for a notable part of the year. With a PDC threshold of 80%, patients classified as being nonadherent did not have their DOAC stocked at home for at least 73 days of the year. Additionally, the presence of medication at home does not guarantee (proper) intake. Thus, it is reasonable to assume that the actual prevalence of AF patients at risk of thromboembolic events due to inappropriate DOAC intake is likely higher. Therefore, the topic of therapy adherence, particularly among patients at risk of becoming nonadherent, warrants special attention in the outpatient setting.

### Predictors of 1-Year Nonadherence

While some studies, including our own, reported that men are more likely to be nonadherent, the majority of earlier performed studies did not report any sex-related differences in adherence or even elucidated female sex as a predictor for nonadherence. 12 30 31 Previous studies have shown that women with AF are older, have more comorbidities, and have a greater risk of thromboembolic events. 32 This apparent sex-related difference could be, partially, attributed to these factors instead, as comorbidities and a higher stroke risk have been found to be associated with higher adherence. 28 Moreover, findings from prior reports suggest that men may have a four times increased risk of poor self-care compared to women, which may impair adherence to DOAC therapy as well. 33

In accordance with existing literature, twice-daily compared with once-daily dosing regimens and the two corresponding DOACs apixaban and dabigatran were associated with an increased prevalence of nonadherence. 12 13 14 15 16 17 23 24 Nonadherence is often unintentional, and it is understandable that the risk of forgetting a dose is higher with a twice-daily dosing regimen. 34 This was previously reported in patients prescribed other cardiovascular medication as well, where adherence declined with increasing number of doses per day. 35 Another explanation for nonadherence in patients prescribed dabigatran could be the presence of adverse effects, mainly dyspepsia, which is reported to occur more often in patients using this specific DOAC. 2 36

A reduced or lower DOAC dose was another predictor of nonadherence. Depending on the DOAC prescribed and the guideline adhered to, dose reduction is recommended in patients with a history of bleeding. It is reasonable to hypothesize that such patients are more aware of the risk of bleeding complications associated with anticoagulant therapy, and even minor bleeding complications may make these patients hesitant to take their DOAC as prescribed. Additionally, patients who are prescribed a reduced or lower dose, mostly based on comorbidities, may be more likely to experience intercurrent hospital admissions, which can lead to gaps in the uptake of medication and (inadvertent) higher rates of nonadherence.

Moreover, younger patients were found to be less adherent to treatment in our study, consistent with previous research. 8 29 34 This finding is surprising, given that the elderly population is often assumed to be more susceptible to forgetfulness of medication intake. However, comorbidities and thromboembolic complications of AF are more prevalent in patients over 60 years of age. 37 Thus, older patients may have a greater appreciation for the beneficial effects of oral anticoagulant therapy compared to younger patients, who may be relatively free of comorbidities and less aware of the risks of nonadherence. Additionally, polypharmacy is more common among patients aged 60 years or older. These patients may therefore be more accustomed to taking medication; medication might play a greater role in their lives as compared with patients who do not have concomitant drug use. In line with this, there is evidence that the earlier mentioned dosage related decline in adherence becomes less apparent with increasing age. 35 Both increasing age and polypharmacy might be factors supporting the implementation of measures to improve medication management and therapy adherence, such as enhanced social control, medication rolls, and pillboxes. In our study, however, data on supportive measures for medication intake were not available.

### Strengths and Limitations

The population-based study design allowed us to investigate adherence in a large population of unselected participants receiving a DOAC. The main strengths of our study are its large sample size, the availability of a unique patient identifier providing individual patient linkage between different affiliated pharmacies, and the selection of patients with a treatment indication for at least 1 year. In contrast to most previous studies, dispensing information remained available even when patients switched pharmacies. Consequently, our study's dispensing data and derived adherence calculation were comparatively more accurate than those of prior investigations. Additionally, excluding patients with temporary indications for anticoagulant therapy allowed us to focus on nonadherence over a 1-year period among patients with an actual treatment indication for at least 1 year. Lastly, by excluding data of pharmacies that failed to provide uninterrupted data for the entire study duration, we increased the validity of our results.

However, our study has also limitations. First, adherence was based on dispensing data and PDC. On one hand, medication refill does not equate to medication consumption, hence missing doses may remain unnoticed. On the other hand, a gap between refills was automatically regarded as sub-optimal medication intake; reasonable explanations such as peri-operative discontinuation or an intercurrent hospital admission were not taken into account. Therefore, adherence could have been both overestimated and underestimated. However, other available nonadherence measurement tools, such as adherence questionnaires, have disadvantages as well, as they rely on subjective patient-reported nonadherence rates. Similarly, announced “pill counting” methods can disrupt daily practice and potentially lead to overestimation of adherence due to socially desirable behavior or “pill dumping.” The PDC measurement tool allowed us to objectively assess therapy nonadherence in daily clinical practice within a near-nationwide cohort. Moreover, it is important to note that the PDC, along with the 80% threshold, is endorsed as the standard and most appropriate method for medication adherence calculation by the Pharmacy Quality Alliance. Therefore, this approach has been widely used in other studies within this field, including research involving patients treated with DOACs. 38 Second, it is worth noting that the pharmacy database used in our study did not contain data regarding the reasons for nonadherence or clinical outcomes, such as ischemic events. Nonetheless, understanding the underlying causes and potential clinical implications of nonadherence is of utmost significance, and we recognize it as a valuable avenue for future research. Additionally, patients without an indication for DOAC treatment for at least 1 year could have been included in this study. Theoretically, patients undergoing an electrical cardioversion or ablation at the start and at 12 months, without an indication for anticoagulant therapy in between, did fulfill inclusion criteria for the current analysis. This could have resulted in lower estimated than actual adherence. Lastly, direct data on the primary indication for DOAC treatment were unavailable. Instead, a decision tree based on dosing regimens approved for AF was used. Even though the criteria were most sensitive to patients with AF, we cannot rule out that we may have excluded some patients being treated with a DOAC because of AF or included some patients with a different indication in our analyses.

## Conclusion

In this near-nationwide cohort of AF patients newly initiated on a DOAC for at least 1 year, the prevalence of 1-year nonadherence was low yet relevant. Male sex, younger age, a twice daily dosing regimen, treatment with apixaban or dabigatran, and a reduced DOAC dose were independent predictors of nonadherence. These predictors may help identify patients at risk for becoming nonadherent. In order to reduce thromboembolic complications, interventions to reinforce adherence, such as recurrent counseling sessions and medication-taking reminders, might be specifically targeted at these patients.

## Notes

**What Is Known about This Topic?**

- Among patients with atrial fibrillation (AF) who are prescribed direct oral anticoagulants (DOACs), previous studies have reported highly variable rates of nonadherence within the first year of treatment (5–59%).
- There is a gap in current research on DOAC nonadherence in patients with AF, as most prior studies have not accounted for the indicated treatment duration when assessing 1-year nonadherence rates.

**What Does This Paper Add?**

- In this near-nationwide study, the prevalence of 1-year nonadherence was 6.5% among patients with AF newly prescribed a DOAC and receiving at least 1 year of treatment.
- Independent predictors for 1-year nonadherence were: male sex, younger age, a twice daily dosing regimen, treatment with apixaban or dabigatran, and a reduced or lower DOAC dose.
- Due to therapy nonadherence a relevant proportion of patients with AF remain at risk of thromboembolic events for a notable part of the year. We recommend that clinicians give special attention to this issue in the outpatient clinic.
