# Supplementary material for: Prevalence and Predictors of Nonadherence to Direct Oral Anticoagulant Treatment in Patients with Atrial Fibrillation
Source: TH Open. 2023 Sep 27;7(3):e270–9. doi: 10.1055/a-2161-0928 (PMC10533218; doi:10.1055/a-2161-0928)
Supplement: Supplementary file 1 — Supplementary Material [file 10-1055-a-2161-0928-s23060022.pdf]

Supplementary Material

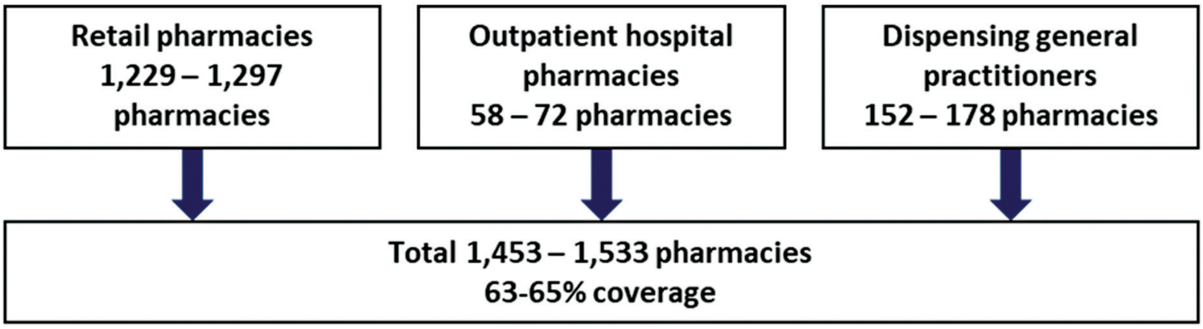

Supplementary Fig. S1 Overview of pharmacies of the IQVIA prescription database. Coverage is expressed as percentage of the total Dutch population and varies per year (2013–2017).

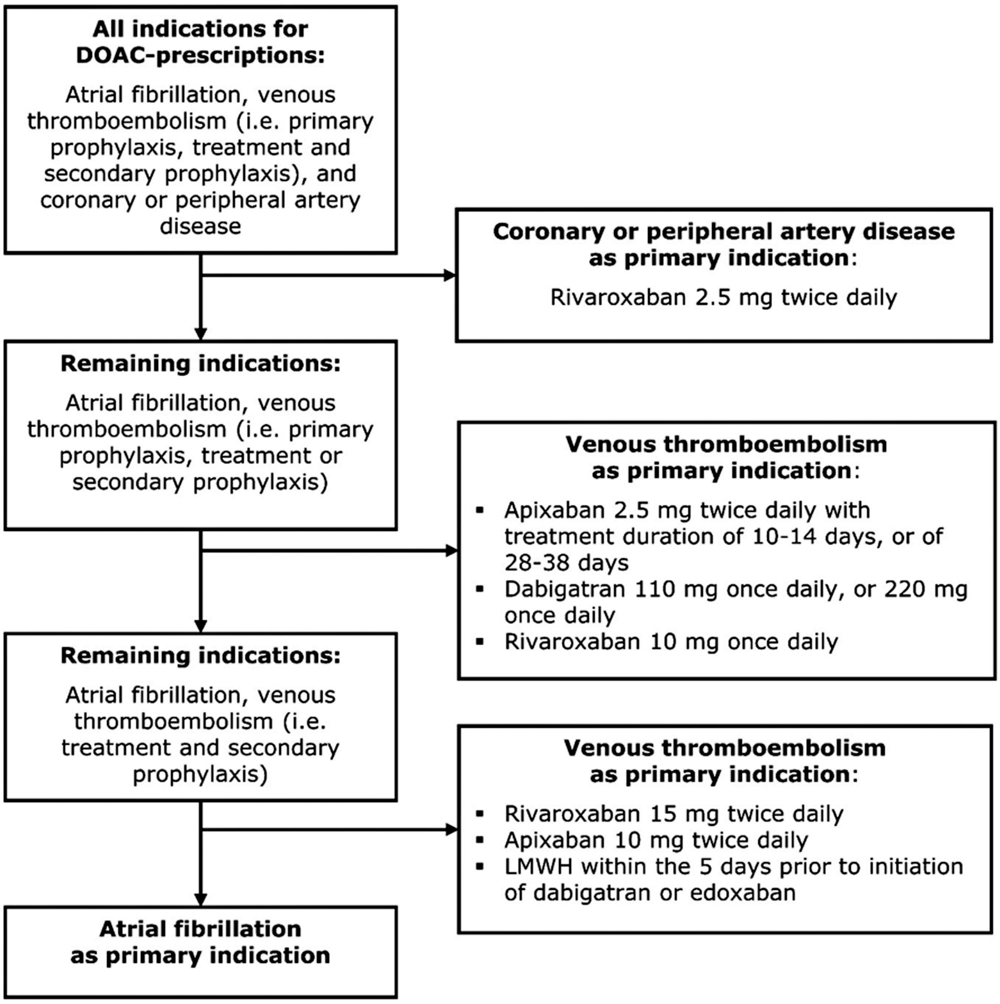

Supplementary Fig. S2 Decision tree to estimate the indication for the initially collected direct oral anticoagulant prescription. DOAC, direct oral anticoagulant; LMWH, low-molecular-weight heparin.

 © 2023. The Author(s).

Supplementary Table S1 Dosing regimens of direct oral anticoagulants (DOACs) approved for atrial fibrillation

Type of DOAC	Standard dose	Reduced dose	Lower dose
Apixaban	5 mg twice daily	2.5 mg twice daily	N.a.
Dabigatran	150 mg twice daily	N.a.	110 mg twice daily
Edoxaban	60 mg once daily	30 mg once daily	N.a.
Rivaroxaban	20 mg once daily	15 mg once daily	N.a.

Note: Dosing regimens in accordance with the European Heart Rhythm Association Practical Guide.[1]

References

1 ESC guideline novel oral anticoagulants for atrial fibrillation. 2021. Accessed October 17, 2022 at: https://www.escardio.org/Guidelines/Recommended-Reading/Heart-Rhythm/Novel-Oral-Anticoagulants-for-Atrial-Fibrillation

 © 2023. The Author(s).
